# Review of "*Ticks: Biology, Disease and Control" *by Alan Bowman & Patricia Nuttall (eds.)

**DOI:** 10.1186/1756-3305-2-1

**Published:** 2009-01-05

**Authors:** Alan R Walker

**Affiliations:** 1 Royal (Dick) School of Veterinary Studies, Summerhall Place, Edinburgh, EH9 1QH, UK

## Review

This fine book demonstrates how far the field of tick biology has progressed in the last 40 years. I remember discussions with colleagues who were studying ticks and associated diseases, whilst I was studying insects as virus vectors, all in a laboratory in Kenya. They claimed their job was much tougher because the textbooks they had to help them consisted of one each from Yuri Balashov, Don Arthur and Harry Hoogstraal; whilst entomology texts could then fill a small library. Apart from a dozen recent texts on various aspects of tick biology we now have Daniel Sonenshine's comprehensive explanatory textbook and this wide multi-author compilation of specialist topics edited by Alan Bowman and Pat Nuttall. It originated in a commissioned special issue of *Parasitology *in 2004, now expanded by further authors and revisions. So unlike many books derived from conference proceedings, 51 well established researchers have written 21 substantial review articles, all supported by long lists of recent literature. The coverage is good, so it is easier to indicate topics not covered directly: gut and digestion, and behaviour are missing; and tick associated pathogens comprise one review on viruses, two on one bacterium each, and two on one genus of protozoa each. Of course, any attempt to cover the gamut of pathogens requires another full textbook, so some generic examples are appropriate for this book. Furthermore, the coverage is a good reflection of current research interests and so it must be emphasized here that this is a book intended for researchers rather than non-specialists seeking facts about the basics of tick biology.

Why study ticks? The standard answer is because they are economically and socially important as agents of disease. The corollary being that studying them is important. But ask a farmer in Uganda what she does about ticks on her Friesian diary herd – once a week dip in organophosphate! Or try sitting on a public health advisory board writing pamphlets about Lyme disease – wear long trousers tucked into socks! And if that is not discord enough, consider the increasing ethical problem of studying ticks. Even understanding their ecology needs information derived from feeding ticks on experimental animals – a nasty business indeed. Resolutions to this dissonance can be found in this book. Improvements in avoiding, preventing and curing vector associated diseases are at least as likely to derive, very unpredictably, from a broad advance in deep understanding, as they are from any specific applied study.

Ticks are not insects, and the great clarification of their population dynamics in relation to pathogen transmission is a major advance for vector biology in general. Tick salivary glands are wonderful subjects for anatomical, physiological and biochemical study and as a source of pharmacological agents they are a deep and rich mine. The ability of ticks to evade host immune resistance provides routes for advances in fundamental immunology as well as inspiration for pharmaceuticals for human and animal treatment. Similarly the extraordinary adaptations of tick transmitted pathogens to exploit these immune evasions, or the normally functioning dermal hypersensitivity reactions to ticks, has greatly increased our understanding of the biology of viruses, bacteria and protozoa. The natural nidality of vector associated pathogens is strongly exemplified by tick spatial distributions. So the advent of geographical information systems, coupled with studies on microclimatic factors on tick survival, has consolidated understanding of a field of such public concern that we now get news of it on television and in newspapers. That is: climate change and its threats. These topics are a selection from this book.

But, inevitably and sadly for those romantics amongst us, the best tick is a dead tick. Thus it remains vital that the essential role of conventional acaricide use remains appreciated; and scope for improvements on it, such as biological agents, manipulations with pheromones, and anti-tick vaccines, continue to be actively pursued at the applied end of a tick research front. The anti-tick vaccine of course remains a star in the tick biologist's storybook. As a final example of what I have been promoting it could not be better. The method of manufacturing specific proteins by the recombinant nucleic acid route was first used to great commercial effect with insulin. The method was invented when a small group of university researchers realized the new understanding of plasmids and how to use them for pure genetics studies could be combined with the new understanding of how DNA works to code for proteins. Structure of DNA can be traced back many decades to studies of how to use X-rays to analyse the three dimensional folding of proteins. To summarize, this book is a must-buy for every tick biologist, and library of every veterinary and medical and biology department on the globe. It is a pleasure to see Cambridge University Press produce it with their exemplary style.

## Competing interests

The author declares that they have no competing interests.

